# Supplementary figures and images for: Murine lupus is neutrophil elastase-independent in the MRL.Faslpr model
Source: PLoS One. 2020 Apr 3;15(4):e0226396. doi: 10.1371/journal.pone.0226396 (PMC7122749; doi:10.1371/journal.pone.0226396)

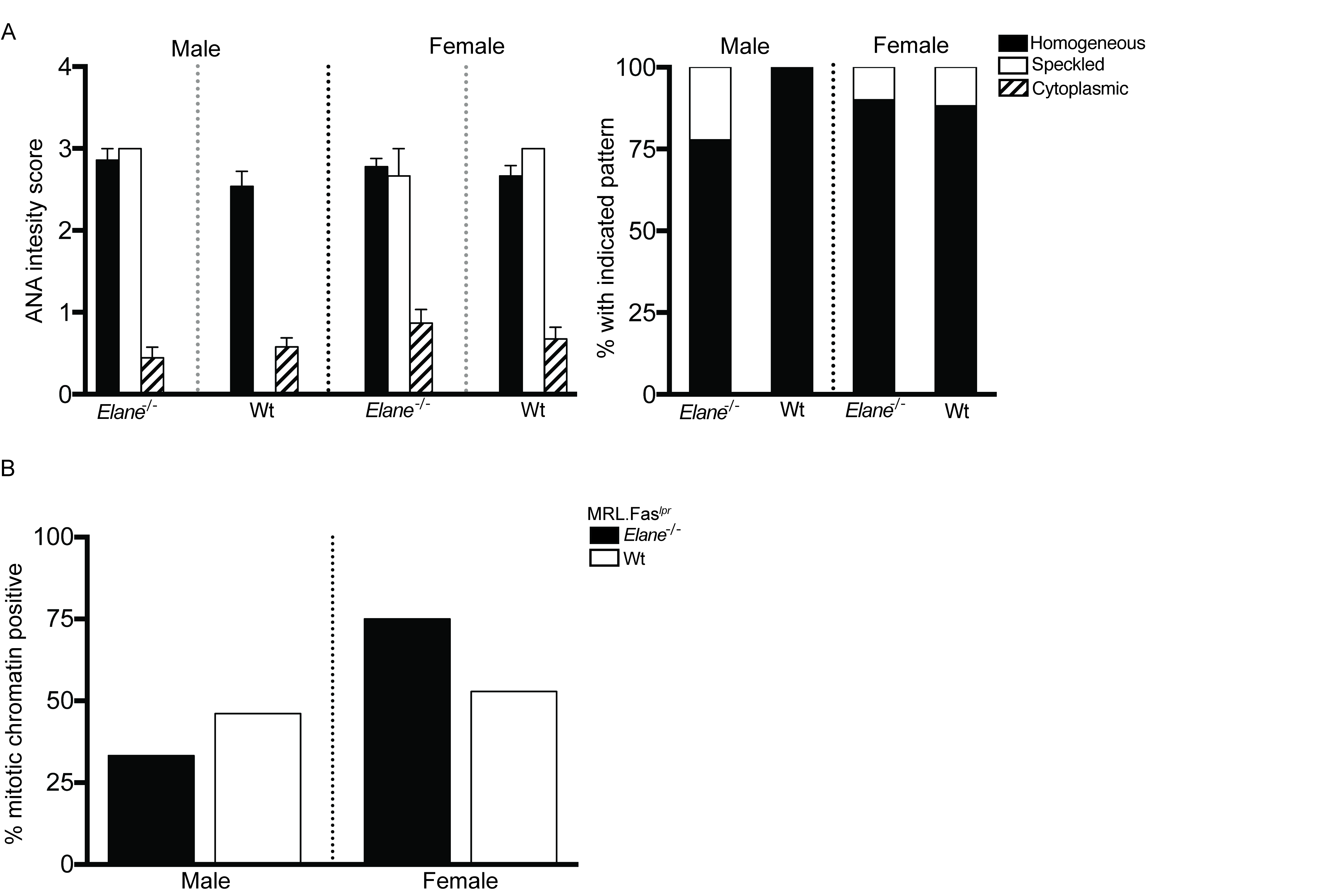

Supplement: S1 Fig — (A) HEp2-ANA slides were scored for intensity of nuclear and cytoplasmic staining patterns (left panel). Data representation and statistics are as in Fig 2F unless otherwise indicated. Dominant ANA pattern classified as nuclear (homogenous), nuclear (speckled), or cytoplasmic (right panel). (B) HEp2-ANA slides were scored for the presence or absence of mitotic chromatin staining. In panel B, a Fisher Exact test was performed to determine statistical significance within each gender (elane-/- males n = 9; elane+/+ males n = 13; elane -/- females n = 20; elane+/+ females n = 17 mice per group). (TIF) [file pone.0226396.s001.tif]
